# Digital dating engagement among young users: gender differences in Tinder use motivations and associations with sexual desire but not self-esteem

**DOI:** 10.3389/fpsyg.2025.1659760

**Published:** 2026-01-02

**Authors:** Pantxika Victoire Morlat, Maria Limniou, Laurence Alison

**Affiliations:** Department of Psychology, University of Liverpool, Liverpool, United Kingdom

**Keywords:** Tinder, online dating, dating application, sexual desire, self-esteem, young users, digital risk awareness, evolutionary mating theory

## Abstract

**Introduction:**

With over 60 million active users worldwide, Tinder is one of the most widely used dating applications. While previous research has associated Tinder use with lower self-esteem, findings have been inconsistent, and little is known about how Tinder use directly relates to sexual desire or the specific motivations that drive young users to use the application.

**Materials and methods:**

The study examined the relationships between Tinder use, user motivations, gender identity, sexual desire, and self-esteem among 305 participants aged 18-30. Participants were categorized as high, medium, or low Tinder users and completed an online survey including the Rosenberg Self-Esteem Scale, the Sexual Drive Inventory-2, and demographic questions.

**Results:**

A X^²^ test of independence revealed significant gender differences in Tinder use motivations (*p* < 0.001), with men seeking casual sex and women more often pursuing serious relationships. A multivariate analysis of variance indicated that Tinder use significantly affected sexual desire subscales, particularly dyadic partner desire (*p* < 0.001). A simple linear regression showed that increased Tinder use was associated with higher overall sexual desire (*p* < 0.001). No significant differences in self-esteem were found across Tinder use groups according to Kruskal-Wallis H test (*p* = 0.92).

**Conclusion:**

These findings highlight sexual desire and relational motivations as central components of Tinder engagement among young adults. The absence of a significant link with self-esteem challenges prior assumptions and underlines the need for more nuanced, longitudinal research. By clarifying behavioral patterns and psychological correlates of dating application use, this study contributes to the growing field of digital relationship research. It also offers insights relevant to mental health support, user education, and platform design.

## Introduction

Since its launch in 2012, Tinder has become one of the most widely used dating applications globally (Rochat et al., 2019). As of 2024, Tinder reported over 60 million monthly active users, with the United Kingdom ranking second in user numbers behind the United States (Tafradzhiyski, 2025). 60.0% of Tinder users are under 35 and 40.0% are between 18 and 24 years old (Tafradzhiyski, 2025), making it a salient platform for examining dating behaviors among young adults.

Tinder’s design facilitates both short-term and long-term relational pursuits, reflecting broader shifts in dating behaviors and culture. While some studies suggest men report higher interest in casual (short-term) sex and display greater sexual attractiveness (Gray and Garcia, 2013; Mealey, 2000), recent research has highlighted that women’s engagement in ‘hookup’ culture^1^ is also increasing (Martino et al., 2024; Prior et al., 2025). Dating applications may play a role in enabling these behaviors (Kettrey et al., 2024). Within evolutionary psychology, mating strategies are theorized to vary by gender and context, with men generally favoring short-term mating more than women (Abramova et al., 2016; Buss and Schmitt, 1993). However, these propositions remain debated and context dependent (Bovet, 2019; Takayanagi et al., 2024). Recent studies have begun to explore how such strategies manifest in digital environments, including Tinder, by analyzing user profiles, preferences, and motivations (Gale et al., 2024; Konings et al., 2024). Two recent comparative studies provide specific evidence on how motivations differ across applications and user groups. Menon (2024) found six primary motivations (i.e., love, ease of communication, distraction, sexual experience, socializing, and trendiness) and reported that Tinder users tended to endorse more casual, entertainment-oriented motivations compared to users of other dating applications. Therefore, Menon (2024) highlights both application-level affordances and life-position indicators as important drivers of use. Hawkins and DeLuca Bishop (2025) similarly analyzed emerging adults across Tinder and other dating applications, and reported that thrill of excitement, love, and trendiness were prominent motives overall, but that casual-sex motivations were more likely among Tinder users. Both these studies indicate that motives may vary by application and user characteristics, reinforcing this study’s focus on Tinder. Additionally, a recent review emphasized the need for further work into how sexual motivations and relationship strategies are shaped by online dating platforms (Ponseti et al., 2022). In addition to mating preferences, other studies have examined how Tinder use is related to personality traits, romantic motivations, and deception concerns (Erevik et al., 2020; Sharabi and Caughlin, 2019; Timmermans and De Caluwé, 2017). For example, gender differences in romantic and sexual intentions have been observed, with men more likely to report using Tinder for casual sex (short-term) and women more likely to seek friendships or long-term relationships (Lopes and Vogel, 2017; Sumter et al., 2017). These patterns align with, but do not confirm, evolutionary hypotheses and may also reflect sociocultural influences.

Despite growing interest in Tinder’s psychological impact, limited research has examined its association with sexual desire. Levine (2003) defines sexual desire as the interplay of forces that draw individuals toward or away from sexual behavior. Rochat et al. (2019) found that sexual desire and problematic Tinder use were linked to self-esteem, but their sample included a wide age range and relationship statuses, limiting generalizability to younger users. Given that younger adults tend to engage in more transient relationships than older adults (de Oliveira et al., 2023; Vasilenko and Lanza, 2014), this study focuses on young adult users aged 18–30 to explore whether sexual desire is associated with frequent Tinder engagement. This age group also aligns with other research on Tinder (e.g., Menon, 2024; Rönnestad, 2017).

Self-esteem is another psychological construct relevant to dating application engagement. While some studies report lower self-esteem among Tinder users compared to non-users (Strubel and Petrie, 2017), others suggest that Tinder may be used to enhance self-perception through positive social feedback (Sumter et al., 2017). However, findings remain inconsistent. For example, Rönnestad (2017) found only a weak relationship between increased intensity of Tinder use and decreased self-esteem in young adults, whereas other studies found no significant link between Tinder use and self-esteem but did identify associations with anxiety and depression (Her and Timmermans, 2021; Holtzhausen et al., 2020). These mixed results highlight the need for further investigation into how Tinder use may influence self-esteem, particularly among younger users.

The present study aims to explore the relationships between Tinder use, user motivations, gender identity, sexual desire, and self-esteem among young adults aged 18–30. Drawing on evolutionary mating theory as a guiding framework, while recognizing its contested status, the study examines gender differences in Tinder use motivations, the potential predictive value of Tinder use on sexual desire and self-esteem. By focusing on a demographically relevant population, this study aims to clarify whether frequent Tinder engagement is associated with heightened sexual desire and diminished self-esteem, contributing to ongoing debates about the psychological effects of dating applications.

These aspects led to the formation of the following hypotheses:

*H1*: Men are more likely than women to use Tinder for casual sex, while women are more likely to use it for serious relationships or friendships.

*H2*: Tinder use is positively associated with sexual desire.

*H3*: Tinder use predicts sexual desire levels.

*H4*: Tinder use is negatively associated with self-esteem.

*H5*: Tinder use predicts self-esteem levels.

## Materials and methods

### Participants

A total of 305 young adult participants (aged 18–30) were recruited using volunteer sampling via social media and campus posters. Eligibility criteria included current Tinder use, being between 18 and 30 years old, and fluency in English. Of the sample, 201 participants (65.9%) identified as women, 94 as men (30.8%), 9 as third gender/other (3.0%), and one participant (0.3%) preferred not to answer the question on gender identity.

Sample size was determined using conventional power analysis thresholds (*α* = 0.05, power = 0.80). For a three-group multivariate analysis of variance (MANOVA), detecting a medium effect size (Cohen’s *f* = 0.25) would require approximately 158 participants, and for a simple linear regression (*f^2^* = 0.15), approximately 55 participants. The final sample of 305 exceeded these thresholds, allowing detection of smaller effects (minimum detectable *f* ≈ 0.18). The sample size also aligns with comparable studies in online dating research (e.g., Orosz et al., 2018; Sumter et al., 2017).

Other sociodemographic variables, such as socioeconomic background, relationship status, and sexual orientation, were not included in the questionnaire. Their absence and potential implications are considered in the Discussion section.

Participants with current depressive symptoms or a history of cyber dating abuse were excluded due to the nature of questions related to dating application, sexual desire, and self-esteem. While this decision was made to minimize distress and reduce confounding effects, given the established link between depression and low self-esteem, it may limit ecological validity and generalizability to clinical populations and those with cyber dating abuse experiences. This limitation is acknowledged in the Discussion section.

### Measures

This study employed an online survey that took approximatively 15 minutes to complete, consisting of 27 items, hosted on the web-secure Qualtrics survey platform (www.qualtrics.com). Demographic data included gender identity, age, and Tinder usage patterns. Participants selected their primary motivation from three options: casual sex, serious relationships, or friendships. These categories reflect common usage patterns in prior research on Tinder (Lopes and Vogel, 2017; Sumter et al., 2017). Tinder engagement was measured via self-reported weekly usage, categorised as ‘low users’ (1-4 hours/week), medium users’ (5-8 hours/week), and ‘high users’ (≥ 9 hours/week). These usage categories are consistent with prior studies, which have classified individuals engaging with digital media for under 5 hours per week as light users (Twenge and Campbell, 2019), around 9 hours per week as heavy users (Hosein, 2005), and approximately 2 hours per day as heavy social media users (You et al., 2023).

The following formula was used to convert the number of hours spent per week on Tinder to the average daily usage in minutes:


$$\frac{\text{Number of hours}\;\mathrm{per}\;\text{week}}{7\;\text{days}}\approx x\;\text{hours}/\mathrm{day}$$

x×60=minutesperday

#### Rosenberg Self-Esteem Scale (RSES)

The RSES (Rosenberg, 1965) was used to measure participants’ self-esteem. It comprises 10 items (five positively worded and five negatively worded) rated on a 4-point Likert scale ranging from 1 (“Strongly agree”) to 4 (“Strongly disagree”). Higher scores indicate greater self-esteem. The RSES was selected because it demonstrated good reliability across different populations, such as Latino populations (α = 0.79; Supple et al., 2013) and showed strong validity in diverse samples, including minors with mild intellectual disabilities (Syropoulou et al., 2021). In the present study, the RSES demonstrated adequate internal reliability (α = 0.75).

#### Sexual Drive Inventory-2 (SDI-2)

The SDI-2 (Spector and Carey, 2001) was used to measure participants’ sexual desire. The SDI-2 was used to measure sexual desire. It includes 14 items divided into three components: dyadic partner desire (DPD), dyadic attractive-person desire (DA-PD), and solitary sexual desire (SSD). Most items were rated on a 9-point Likert scale ranging from 0 to 8, whereas the three items assessing frequency (i.e., items beginning by “[…] how often”) were rated on an 8-point Likert scale ranging from 0 to 7. The response categories differed according to the type of question, including ratings of strength (from “No desire” to “Strong desire”), relevance (from “Not at all important” to “Extremely important”), frequency (from “Not at all” to “More than once a day”), and duration (“Forever” to “Less than one day”). Higher scores indicate higher levels of sexual desire. The SDI-2 was chosen because of its good internal reliability for DPD (α = 0.88), DA-PD (α = 0.84), and SSD (α = 0.92), as well as good test-retest reliability (Wieczorek et al., 2022). The SDI-2 also demonstrated excellent validity across diverse populations, such as a large international cross-cultural study involving 82,243 participants from 42 countries (Castro-Calvo et al., 2024). In this study, the SDI-2 showed strong internal reliability (α = 0.90).

### Procedure

**Figure 1:**
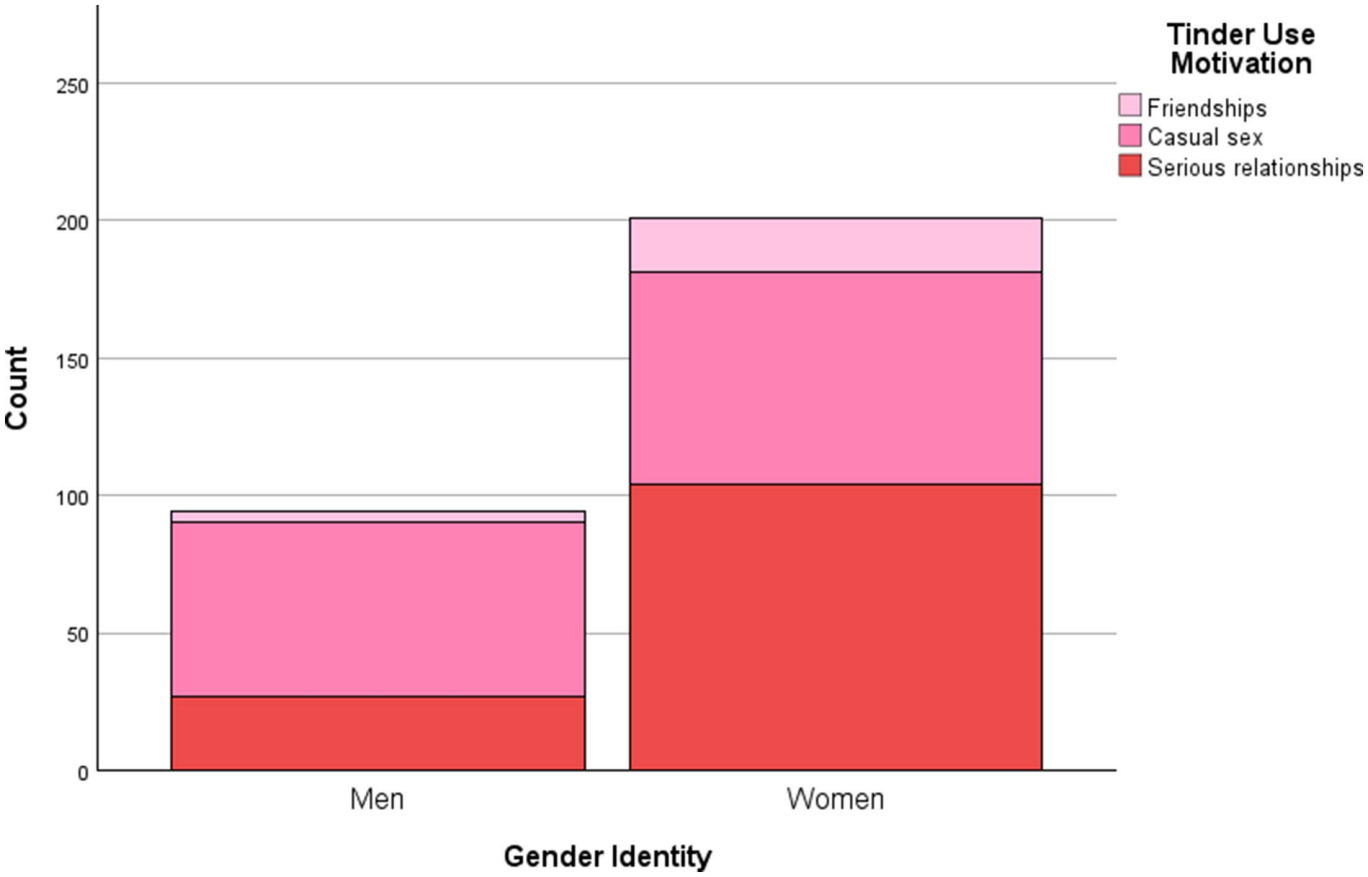
Frequency of Tinder Use Motivations by Gender Identity.

Participants completed the anonymous online survey after reviewing an electronic information sheet and providing informed consent. Given the sensitive nature of some items, participants were provided with links to mental and sexual health resources (e.g., Online Dating Association). Upon completion, a debriefing form with contact details for support services was provided (e.g., Young Minds) (see Table 1 and Figure 1).

**Table 1:** Cross tabulation of gender identity and tinder use motivations.

	Friendships (%)	Casual sex (%)	Serious relationships (%)
Man	4.3%	67.0%	28.7%
Women	10.0%	38.3%	51.7%
Third gender/other	0.0%	100.0%	0.0%
Prefer not to say	0.0%	100.0%	0.0%

#### Group data analysis

Despite the number of participants who preferred not to disclose their gender identity and those who identified as a third gender or other were few in number (*n* = 1 and *n* = 9, respectively), they were retained in analyses to ensure inclusivity and to avoid exclusion bias. Excluding these participants would not only erase a segment of the population but could also distort parameter estimates by conditioning analyses on an incomplete representation of gender diversity (Fafchamps and Labonne, 2017; Lipsky, 2010). Including all gender identities, even with limited statistical power for subgroup-specific inference, aligns with best practices for equity, transparency, and the accurate reflection of social realities (American Psychological Association, 2020). While results pertaining to this subgroup should be interpreted with caution due to the small sample size, their inclusion supports the broader movement toward more representative and ethical research practices. Future research with larger, gender-diverse samples is needed to explore these patterns more robustly. This limitation is discussed in the final section to avoid over-interpretation.

## Results

Data were analyzed using the Statistical Package for the Social Sciences (SPSS) version 29. Descriptive statistics (means and standard deviations) and inferential statistics (X^²^ test of independence, MANOVA, simple linear regression, and Kruskal–Wallis H test) were conducted to examine the five hypotheses. For all analyses, the significance level was fixed at *p* < 0.05. Less than 5% of cases had missing data, and no systematic patterns were observed. Missing data were excluded using listwise deletion.

### *H1*: Gender identity and Tinder use motivations

A X^²^ test of independence revealed a significant association between gender identity and Tinder use motivations, X^²^ (6, 305) = 31.96, *p* < 0.001, Cramer’s *V* = 0.23, indicating a moderate effect. Men were more likely to use Tinder for casual sex, while women favoured more serious relationships and friendships compared to men, supporting *H*1 (see Table 1; Figure 1). Participants identifying as third gender/other or preferring not to disclose their gender exclusively selected casual sex as their primary motivation. However, due to the small sample size in these subgroups (*n* = 9 and *n* = 1, respectively), these findings should be interpreted cautiously.

### *H2*: Tinder use and SDI-2

Box’s test of equality of covariance matrices, Levene’s test of equality of variances, and the Shapiro–Wilk test of normality were all nonsignificant, indicating that the assumptions of homogeneity of covariance matrices, equality of variances, and normality were met. A MANOVA revealed a significant multivariate effect of Tinder use on SDI-2 subscales, Pillai’s Trace = 0.141, *F* (6, 572) = 7.22 *p* < 0.001, *ηp*² = 0.07, indicating a medium effect. Follow-up univariate analyses showed significant effects of Tinder use on DPD, *F* (2, 287) = 20.87, *p* < 0.001, *ηp*² = 0.13 (medium effect); DA-PD, *F* (2, 287) = 6.88, *p* = 0.001, *ηp*² = 0.05 (moderate effect); and SSD, *F* (2, 287) = 12.25, *p* < 0.001, *ηp*² = 0.08 (medium effect). The strongest association was observed for DPD (*M* = 29.89, *SD* = 8.03; see Table 2). These findings support *H*2; however, they are correlational and do not imply causality.

**Table 2:** Mean (M) and standard deviation (SD) for DPD, DA-PD, and SSD by Tinder Use.

	Tinder use	M	SD
DPD	Low	29.02	7.56
	Medium	34.00	7.94
	High	42.82	5.84
	Total	29.89	8.03
DA-PD	Low	10.30	3.53
	Medium	11.65	2.74
	High	13.90	2.77
	Total	10.53	3.53
SSD	Low	23.59	8.82
	Medium	26.65	7.46
	High	36.64	11.36
	Total	24.30	9.17

DPD, dyadic partner desire; DA-PD, dyadic attractive-person desire; SSD, solitary sexual desire (Spector and Carey, 2001).

### *H3*: Tinder use as predictor of SDI-2

**Figure 2:**
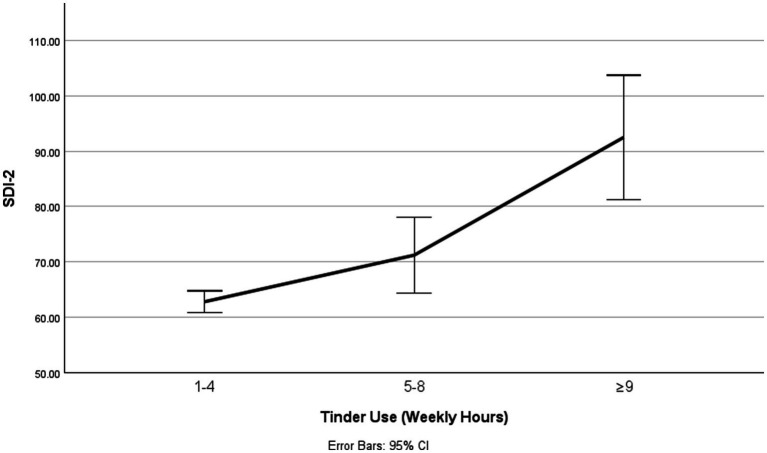
Relationship Between Weekly Hours of Tinder Use and SDI-2. SDI-2 = Sexual Desire Inventory-2 (Spector and Carey, 2001).

All relevant assumptions for regression analyses were tested and met, including normality of residuals, homogeneity of variance, and absence of multicollinearity. A simple linear regression analysis showed that Tinder use was significantly associated with SDI-2, adjusted *R*² = 0.11, *β* = 0.33, *F* (1, 303) = 37.80, *p* < 0.001, *f* ² = 0.12, indicating a moderate effect. A positive association was found between Tinder use and SDI-2, *B* = 13.02, *SE* = 2.12, *t* (303) = 6.15, *p* < 0.001, 95% CI [8.85, 17.19]. Each category increase in weekly Tinder use (1-4 hours → 5-8 hours → ≥ 9 hours) corresponded to a 13-point increase in SDI-2 scores. While statistically robust, the effect size is modest and should be interpreted accordingly. These results support *H*3 in terms of association, but causal inference is not warranted due to the cross-sectional design (see Table 2 and Figure 2).

### *H4* and *H5*: Tinder use and RSES

A Kruskal-Wallis H test was conducted to examine differences in RSES across Tinder use groups, as the RSES scores were not normally distributed. No significant differences were found across groups (*p* = 0.92), leading to the rejection of *H*4 and *H*5. While mean self-esteem scores were relatively stable across usage groups, the variability within groups, particularly among medium users (*M* = 22.48, *SD* = 6.01; see Table 3), was notable. This inconsistency, combined with prior mixed findings in the literature, suggests that the relationship between Tinder use and self-esteem may be more complex than hypothesized and warrants further investigation.

**Table 3:** Mean (M) and Standard Deviation (SD) for SDI-2 and RSES by Tinder use.

	Tinder use	M	SD
SDI-2	Low	62.79	16.76
	Medium	71.21	15.10
	High	92.54	17.70
	Total	64.54	17.71
RSES	Low	21.91	4.55
	Medium	22.48	6.01
	High	21.83	9.52
	Total	21.95	4.91

SDI-2, Sexual Desire Inventory-2 (Spector and Carey, 2001); RSES, Rosenberg Self-Esteem Scale (Rosenberg, 1965).

## Discussion

This study explored young adults’ motivations for using the Tinder application for online dating and examined associations between Tinder use, sexual desire, and self-esteem, drawing on evolutionary mating theory. Five hypotheses were tested using X^²^ test of independence, MANOVA, simple linear regression, and Kruskal–Wallis H test. While evolutionary psychology has been widely applied to offline dating contexts, its relevance to digital platforms remains underexplored. This study contributes to this gap by examining how mating motivations and psychological traits manifest in a swipe-based environment.

Findings of this study revealed gender differences in Tinder use motivations, with men more likely to report seeking casual sex and women more inclined toward serious relationships and friendships. These patterns align with prior research and evolutionary hypotheses suggesting differential mating strategies (Abramova et al., 2016; Hawkins and DeLuca Bishop, 2025; Menon, 2024). However, it is important to interpret these findings with nuance. While aggregate trends may reflect broader social norms, individual motivations are diverse and context dependent. For example, some women may pursue casual encounters, and some men may seek committed relationships, highlighting the need to avoid reinforcing binary stereotypes. From a feminist poststructuralist perspective, these patterns may reflect gendered expectations around sexuality and emotional investment (Siegel and Meunier, 2019). Women’s experience of disappointment or mismatch may arise not only from interpersonal dynamics but also from broader cultural scripts that shape dating expectations. Estimating gender differences is complex, and further research is needed to establish more conclusive findings.

Users may see Tinder as a self-enhancement tool, but unmet expectations may result in interpersonal conflict and diminished self-esteem (Orosz et al., 2018). Nonetheless, contrary to some prior research, this study found no significant association between Tinder use and self-esteem. This null result is noteworthy and suggests that frequent Tinder engagement may not uniformly impact users’ self-perceptions. While descriptive data showed slightly lower self-esteem among high-frequency users, the differences were not statistically significant. This may reflect resilience factors among younger users, such as digital literacy (Park and Kim, 2024). The absence of a measurable effect also emphasizes the multifaceted nature of self-esteem. Individual differences, psychological traits, and contextual factors likely moderate the relationship between application use and self-perception (Khandelwal and Bhambri, 2024; Khamassi et al., 2025). Moreover, the cross-sectional design limits the ability to detect cumulative or delayed psychological effects. These findings highlight the importance of treating null results as informative and suggest that future research should examine self-esteem trajectories over time.

This study found a positive association between Tinder use and sexual desire, particularly DPD. Users who spent more time on Tinder reported higher sexual desire scores, consistent with evolutionary mating theory’s emphasis on perceived mating opportunities (Buss, 2008). Matching with others, even without meeting in person, may enhance users’ sense of desirability and stimulate sexual interest. While increased Tinder use correlated with higher sexual desire, causality cannot be inferred. It is also important to avoid conflating sexual desire with sexual behavior or risk. For instance, mismatches in user motivations (e.g., one seeking commitment, the other casual sex) may lead to interpersonal tension, but this study did not assess experiences of coercion or harm. Any discussion of vulnerability or distress must remain framed as a direction for future research.

Taken together, evolutionary and feminist poststructuralist perspectives offer complementary rather than mutually exclusive lenses for interpreting digital dating behavior. Evolutionary mating theory highlights broad, adaptive tendencies that may shape patterns of mate-seeking and sexual interest, while feminist poststructuralist approaches emphasize how those tendencies are mediated and performed within gendered cultural contexts and power relations (Buss and Schmitt, 2011). Integrating these perspectives encourages careful interpretation of population-level trends without reifying stereotypes at the individual level, and it directs attention to how social norms and interactional dynamics shape the expression of evolved predispositions on platforms like Tinder.

Several limitations should be considered when interpreting these findings. The study may be affected by self-selection bias and may not represent individuals from diverse social or environmental backgrounds. Participants were young adults (18–30 years old), an age group typically associated with lower self-esteem compared to middle adulthood (peak at 50 years old; Orth et al., 2012), which likely influenced the results. The omission of other sociodemographic variables (e.g., socioeconomic background, relationship status, and sexual orientation) limits the generalizability of findings and prevents examination of whether associations between Tinder use, sexual desire, and self-esteem, vary across these important demographic subgroups. The study also lacked information on participants’ duration of Tinder use, as long-term users may be less affected, whereas newer users could experience stronger impacts on sexual desire and self-esteem. Another limitation is how Tinder use was measured as a whole and did not specify different activities (i.e., swiping, messaging, virtual meeting). Future research should adopt cross-cultural designs and consider application usage duration and specificities as a moderating factor. Furthermore, the exclusion of participants with current depressive symptoms restricts the generalizability of the findings to clinical adult populations. Future studies should consider including participants with a mental health diagnosis to determine whether the relationships between Tinder use, self-esteem, and sexual desire are consistent across both clinical and non-clinical samples. Similarly, although excluding participants with prior cyber dating abuse was justified for ethical considerations, this reduces ecological validity. Another aspect is the small sample size representing participants identifying as third gender/other; further research should include larger gender samples to allow for the investigation of statistically significant differences. Future research should incorporate mixed-method approaches, including qualitative interviews and diary studies, to capture the lived experiences of Tinder users. Exploring how motivations, expectations, and psychological outcomes evolve over time would offer richer insights into the role of dating applications in young adults’ relational and emotional lives.

Understanding the associations between Tinder use, user motivations, and sexual desire in young people may offer useful insights for discussions about digital safety and well-being while remaining clear about the limits of the evidence. Broader reports indicate increase in incidents of sexual violence involving online dating platforms, with females disproportionately affected (National Crime Agency, 2022). This study did not measure coercion, harassment, or sexual violence and therefore cannot address risk, harm, or safety outcomes. Prior research has documented that dating applications can facilitate unwanted sexual comments, unsolicited images, and other forms of online harassment that sometimes escalate offline (Echevarria et al., 2022; Zytko et al., 2021). Although such evidence falls outside the scope of this study, it accentuates the continued need for user education and robust platform safeguards in contexts where motivations and expectations may diverge.

This study contributes to the growing literature on online dating by reporting associations between Tinder use, sexual desire, and self-esteem among young adults aged 18 to 30. The findings are consistent with aspects of evolutionary mating theory, with men in this sample being more likely to report motivations associated with short-term sexual encounters and women were more likely to report motivations associated with long-term relationships. These gendered patterns reflect trends and should be interpreted with caution, recognizing substantial individual and potential cultural variation. Tinder use was not significantly associated with self-esteem in this cross-sectional sample, while higher frequency of Tinder use was associated with greater reported sexual desire, particularly DPD. Future research using longitudinal and qualitative designs is needed to test causal mechanisms and to evaluate whether and how Tinder features, user education, or mental health supports influence emotional well-being, relational outcomes, and digital safety over time.

## Data Availability

The raw data supporting the conclusions of this article will be made available by the authors, without undue reservation.

## Footnotes

1 Hookup culture refers to a social environment in which casual, non-committed sexual encounters are normalized, expected, and structured by shared norms and peer dynamics, particularly among young adults (Bogle, 2008).
