# Supplementary material for: Digital dating engagement among young users: gender differences in Tinder use motivations and associations with sexual desire but not self-esteem
Source: Front Psychol. 2026 Jan 2;16:1659760. doi: 10.3389/fpsyg.2025.1659760 (PMC12808411; doi:10.3389/fpsyg.2025.1659760)
Supplement: Supplementary file 1 [file Data_Sheet_1.docx]

**Appendix**: **Psychometric instruments**

**Self-Esteem Scale (RSES; Rosenberg, 1965)**

Please record the appropriate answer for each item, depending on whether you strongly agree, agree, disagree, or strongly disagree with it (1 = Strongly agree; 2 = Agree; 3 = Disagree; 4 = Strongly disagree).

1. On the whole, I am satisfied with myself.

2. At times I think I am no good at all.

3. I feel that I have a number of good qualities.

4. I am able to do things as well as most other people.

5. I feel 1do not have much to be proud of.

6. I certainly feel useless at times.

7. I feel that I'm a person of worth.

8. I wish I could have more respect for myself.

9. All in all, I am inclined to think that I am a failure.

10. I take a positive attitude toward myself.

**Sexual Drive Inventory-2 (SDI-2; Spector & Carey, 2001)**

This questionnaire asks about your level of sexual desire. By desire, we mean interest in or wish for sexual activity. For each item, please select the answer that best shows your thoughts and feelings.

1. During the last month, how often would you have liked to engage in sexual activity with a partner (for example, touching each other’s genitals, giving or receiving oral stimulation, intercourse, etc.)? (0 = Not at all; 1 = Once a month; 2 = Once every two weeks; 3 = Once a week; 4 = Twice a week; 5 = 3 to 4 times a week; 6 = Once a day; 7 = More than once a day)
2. During the last month, how often have you had sexual thoughts involving a partner? (0 = Not at all; 1 = Once a month; 2 = Once every two weeks; 3 = Once a week; 4 = Twice a week; 5 = 3 to 4 times a week; 6 = Once a day; 7 = More than once a day)
3. When you have sexual thoughts, how strong is your desire to engage in sexual behavior with a partner? (From 0 to 8, 0 = No desire and 8 == Strong desire)
4. When you first see an attractive person, how strong is your sexual desire? (From 0 to 8, 0 = No desire and 8 = Strong desire)
5. When you spend time with an attractive person (for example, at work or school), how strong is your sexual desire? (From 0 to 8, 0 = No desire and 8 = Strong desire)
6. When you are in romantic situations (such as a candle-lit dinner, a walk on the beach, etc.), how strong is your sexual desire? (From 0 to 8, 0 = No desire and 8 = Strong desire)
7. How strong is your desire to engage in sexual activity with a partner? (From 0 to 8, 0 = No desire and 8 = Strong desire)
8. How important is it for you to fulfill your sexual desire through activity with a partner? (From 0 to 8, 0 = No at all important and 8 = Extremely important)
9. Compared to other people of your age and sex, how would you rate your desire to behave sexually with a partner? (From 0 to 8, 0 = No desire and 8 = Strong desire)
10. During the last month, how often would you have liked to behave sexually by yourself (for example, masturbating, touching your genitals etc.)? (1 = Not at all; 2 = Once a month; 3 = Once every two weeks; 4 = Once a week; 5 = Twice a week; 6 = 3 to 4 times a week; 7 = Once a day; 8 = More than once a day)
11. How strong is your desire to engage in sexual behavior by yourself? (From 0 to 8, 0 = No desire and 8 = Strong desire)
12. How important is it for you to fulfill your desires to behave sexually by yourself? (From 0 to 8, 0 = No at all important and 8 = Extremely important)
13. Compared to other people of your age and sex, how would you rate your desire to behave sexually by yourself? (From 0 to 8, 0 = No desire and 8 = Strong desire)
14. How long could you go comfortably without having sexual activity of some kind? (0 = Forever; 1 = A year or two; 2 = Several months; 3 = A month; 4 = A few weeks; 5 = A week; 6 = A few days; 7 = One day; 8 = Less than one day)
